# Supplementary material for: The amygdala is not necessary for the familiarity aspect of recognition memory
Source: Nat Commun. 2023 Dec 7;14:8109. doi: 10.1038/s41467-023-43906-8 (PMC10703781; doi:10.1038/s41467-023-43906-8)
Supplement: Supplementary file 1 — Supplementary Information [file 41467_2023_43906_MOESM1_ESM.pdf]

Table S1

ANOVA results for Experiment 2

Predictor	df_{Num}	df_{Den}	F	p	η^2_p	95% CI
Group	1	6	2.61	.157	.30	[0.00, 0.70]
Timepoint	1	6	2.21	.188	.27	[0.00, 0.68]
Type	1	6	63.80	.000	.91	[0.62, 0.96]
Delay	1	6	31.66	.001	.84	[0.38, 0.93]
Group × Timepoint	1	6	3.05	.131	.34	[0.00, 0.72]
Group × Type	1	6	1.73	.237	.22	[0.00, 0.65]
Group × Delay	1	6	0.17	.697	.03	[0.00, 0.47]
Timepoint × Type	1	6	19.44	.005	.76	[0.20, 0.90]
Timepoint × Delay	1	6	0.64	.455	.10	[0.00, 0.56]
Type × Delay	1	6	9.54	.021	.61	[0.01, 0.84]
Group × Timepoint × Type	1	6	14.23	.009	.70	[0.10, 0.88]
Group × Timepoint × Delay	1	6	0.17	.690	.03	[0.00, 0.47]
Group × Type × Delay	1	6	3.00	.134	.33	[0.00, 0.71]
Timepoint × Type × Delay	1	6	0.39	.555	.06	[0.00, 0.53]
Group × Timepoint × Type × Delay	1	6	0.01	.935	.00	[0.00, 0.24]

Note. Test was two sided. df_{Num} indicates degrees of freedom numerator. df_{Den} indicates degrees of freedom denominator. η^2_p indicates partial eta-squared, 95% CI indicates the two-tailed 95% confidence intervals around the partial eta-squared. Group = amygdala damage or unoperated controls, Timepoint = pre- or post-surgery/rest, Type = normal or probe trial, Delay = short or long retention interval.
