# Supplementary material for: The amygdala is not necessary for the familiarity aspect of recognition memory
Source: Nat Commun. 2023 Dec 7;14:8109. doi: 10.1038/s41467-023-43906-8 (PMC10703781; doi:10.1038/s41467-023-43906-8)
Supplement: Supplementary file 3 — Reporting Summary [file 41467_2023_43906_MOESM3_ESM.pdf]

nature portfolio

Corresponding author(s): Basile, Benjamin M.

Last updated by author(s): Nov 13, 2023

Reporting Summary

Nature Portfolio wishes to improve the reproducibility of the work that we publish. This form provides structure for consistency and transparency in reporting. For further information on Nature Portfolio policies, see our Editorial Policies and the Editorial Policy Checklist.

Statistics

For all statistical analyses, confirm that the following items are present in the figure legend, table legend, main text, or Methods section.

n/a	Confirmed	
☐	☒	The exact sample size (*n*) for each experimental group/condition, given as a discrete number and unit of measurement
☐	☒	A statement on whether measurements were taken from distinct samples or whether the same sample was measured repeatedly
☐	☒	The statistical test(s) used AND whether they are one- or two-sided<br>*Only common tests should be described solely by name; describe more complex techniques in the Methods section.*
☐	☒	A description of all covariates tested
☐	☒	A description of any assumptions or corrections, such as tests of normality and adjustment for multiple comparisons
☐	☒	A full description of the statistical parameters including central tendency (e.g. means) or other basic estimates (e.g. regression coefficient) AND variation (e.g. standard deviation) or associated estimates of uncertainty (e.g. confidence intervals)
☐	☒	For null hypothesis testing, the test statistic (e.g. F, t, r) with confidence intervals, effect sizes, degrees of freedom and P value noted<br>*Give P values as exact values whenever suitable.*
☐	☒	For Bayesian analysis, information on the choice of priors and Markov chain Monte Carlo settings
☐	☒	For hierarchical and complex designs, identification of the appropriate level for tests and full reporting of outcomes
☐	☒	Estimates of effect sizes (e.g. Cohen's *d*, Pearson's *r*), indicating how they were calculated

Our web collection on statistics for biologists contains articles on many of the points above.

Software and code

Policy information about availability of computer code

Data collection	Data collected with Presentation (Neurobehavioral Systems; v 20.1) using custom code.
Data analysis	Analyzed using Microsoft Excel (version 2302), R Statistical Software (v 2023.09.1), and the dual process analysis spreadsheet provided online by Dr. Andy Yonelinas (https://yonelinas.faculty.ucdavis.edu/roc-analysis/, last accessed Nov 13, 2023).

For manuscripts utilizing custom algorithms or software that are central to the research but not yet described in published literature, software must be made available to editors and reviewers. We strongly encourage code deposition in a community repository (e.g. GitHub). See the Nature Portfolio guidelines for submitting code & software for further information.

Data

Policy information about availability of data

All manuscripts must include a data availability statement. This statement should provide the following information, where applicable:

- Accession codes, unique identifiers, or web links for publicly available datasets
- A description of any restrictions on data availability
- For clinical datasets or third party data, please ensure that the statement adheres to our policy

The datasets generated during and/or analyzed during the current study are available from the corresponding author on reasonable request.

Human research participants

Policy information about studies involving human research participants and Sex and Gender in Research.

Reporting on sex and gender	N/A
Population characteristics	N/A
Recruitment	N/A
Ethics oversight	N/A

Note that full information on the approval of the study protocol must also be provided in the manuscript.

Field-specific reporting

Please select the one below that is the best fit for your research. If you are not sure, read the appropriate sections before making your selection.

☒ Life sciences ☐ Behavioural & social sciences ☐ Ecological, evolutionary & environmental sciences

For a reference copy of the document with all sections, see nature.com/documents/nr-reporting-summary-flat.pdf

Life sciences study design

All studies must disclose on these points even when the disclosure is negative.

Sample size	Sample size were chosen based on the standard practices of the field and prior studies of lesions in nonhuman primates (e.g., Rudebeck & Murray, 2011, J. Neuro.; Browning et al., 2013, J. Neuro.; Matsumoto et al., 2016, J. Neuro.).
Data exclusions	Data exclusions are described in text. They are: For Experiment 3, 7% of data were excluded because they did not pass the manipulation check. For Experiment 5, 3.4 % of data were excluded for the modeling analysis, but not the other analyses, because they were from sessions in which the modeled parameters did not converge.
Replication	Experiments 1-4 test the same construct using different methods, thus providing conceptual replications of each other. No exact replication of these studies has been attempted.
Randomization	Subjects were randomly assigned to control or experimental groups.
Blinding	Data collection was carried out by automated computerized testing, thus eliminating the potential for unblinded experimenters to influence subject's performance during testing sessions.

Reporting for specific materials, systems and methods

We require information from authors about some types of materials, experimental systems and methods used in many studies. Here, indicate whether each material, system or method listed is relevant to your study. If you are not sure if a list item applies to your research, read the appropriate section before selecting a response.

Materials & experimental systems

n/a	Involved in the study	
☒	☐	Antibodies
☒	☐	Eukaryotic cell lines
☒	☐	Palaeontology and archaeology
☐	☒	Animals and other organisms
☒	☐	Clinical data
☒	☐	Dual use research of concern

Methods

n/a	Involved in the study	
☒	☐	ChIP-seq
☒	☐	Flow cytometry
☒	☐	MRI-based neuroimaging

Animals and other research organisms

Policy information about studies involving animals; ARRIVE guidelines recommended for reporting animal research, and Sex and Gender in Research

Laboratory animals	Adult male rhesus macaques (Macaca mulatta; mean age at start of testing 4.93 years)
Wild animals	The study did not involve wild animals.
Reporting on sex	Data come from males. In accordance with the NIH Policy on Sex as a Biological Variable nonhuman primates represent an "acutely scarce resource" and use of acutely scarce resources provides strong justification for using only one sex.
Field-collected samples	The study did not involve samples collected from the field.
Ethics oversight	This study was carried out in accordance with the Guide for the Care and Use of Laboratory Animals and the US Animal Welfare Act. All procedures were reviewed and approved by the National Institute of Mental Health Animal Care and Use Committee.

Note that full information on the approval of the study protocol must also be provided in the manuscript.
